# Screen Printed Carbon Electrode Based Electrochemical Immunosensor for the Detection of Dengue NS1 Antigen

**DOI:** 10.3390/diagnostics4040165

**Published:** 2014-11-20

**Authors:** Om Parkash, Chan Yean Yean, Rafidah Hanim Shueb

**Affiliations:** Department of Medical Microbiology and Parasitology, School of Medical Science, Universiti Sains Malaysia, 16150 Kubang Kerian, Kelantan, Malaysia; E-Mails: op11_med024@student.usm.my (O.P.); yeancyn@yahoo.com (C.Y.Y.)

**Keywords:** electrochemical immunosensor, dengue diagnosis, NS1, screen printed carbon electrode, streptavidin/biotin

## Abstract

An electrochemical immunosensor modified with the streptavidin/biotin system on screen printed carbon electrodes (SPCEs) for the detection of the dengue NS1 antigen was developed in this study. Monoclonal anti-NS1 capture antibody was immobilized on streptavidin-modified SPCEs to increase the sensitivity of the assay. Subsequently, a direct sandwich enzyme linked immunosorbent assay (ELISA) format was developed and optimized. An anti-NS1 detection antibody conjugated with horseradish peroxidase enzyme (HRP) and 3,3,5,5'-tetramethybezidine dihydrochloride (TMB/H_2_O_2_) was used as an enzyme mediator. Electrochemical detection was conducted using the chronoamperometric technique, and electrochemical responses were generated at −200 mV reduction potential. The calibration curve of the immunosensor showed a linear response between 0.5 µg/mL and 2 µg/mL and a detection limit of 0.03 µg/mL. Incorporation of a streptavidin/biotin system resulted in a well-oriented antibody immobilization of the capture antibody and consequently enhanced the sensitivity of the assay. In conclusion, this immunosensor is a promising technology for the rapid and convenient detection of acute dengue infection in real serum samples.

## 1. Introduction

Dengue continues to be a major public health concern in tropical and subtropical countries. Currently, there are over 3.6 billion people at risk from dengue infection. Every year, it is estimated that 390 million dengue infections occur worldwide [1,2]. This leads to 250,000–500,000 cases of dengue hemorrhagic fever (DHF) with 5%–10% mortality annually [3,4]. Until now, no effective vaccine or antiviral drugs were available to combat this disease. Consequently, early and prompt diagnosis of dengue can help patient triage and care management [5,6]. Routine laboratory methods used for the diagnosis of dengue infection are viral culture, viral nucleic acid amplification by reverse transcriptase PCR (RT-PCR), and serological tests like immunoglobulin M (IgM) capture ELISA and immunoglobulin G (IgG) capture ELISA [7]. The first two tests have limitations since they are expensive and require appropriate lab facilities and well-trained personnel, so they are not feasible in a routine diagnostic laboratory. On the other hand, a serological test such as IgM capture ELISA has limitations: it requires at least five days to achieve a detectable amount of IgM antibodies, while IgG antibodies are detectable after nine days [8,9,10].

Dengue nonstructural 1 (NS1) antigen has been reported to be present at high concentration in serum during the early stage of infection, by using an ELISA-based test. Thus dengue NS1 has been used either in ELISA or rapid diagnostic test (RDT) for early diagnosis of dengue [11,12,13]. However, conventional ELISA is not economical if it is used for a small number of samples [14]. In contrast, rapid diagnostic testing for NS1 detection based on immunochromatography could be used in point-of-care testing [15]. This assay has a lower sensitivity than ELISA. Moreover, it is quite susceptible to unfavorable storage conditions and can give false results [14]. Compared with ELISA and RDTs, biosensors can provide quantitative responses through a transducer in the form of measurable electric signal. In addition, biosensors present enormous advantages such as simpler management, easier miniaturization, and the possibility of on-site monitoring [16]. At present, a few immunosensors have been developed to detect the NS1 antigen using various transducers including optical, piezoelectric, and electrochemical [16,17]. Among the available detection approaches, electrochemical methods are of particular interest owing to their simplicity, accuracy, high sensitivity, and possible compatibility with portable systems suitable for point-of-care testing [18,19].

Recently, several immunosensor devices have been developed on screen-printed carbon electrodes (SPCEs) [20]. The main advantages of the screen printed electrode include simplicity, versatility, modest cost, portability, ease of operation, reliability, small size, and mass production capabilities [21]. For the fabrication of SPCEs, carbon inks are particularly attractive because they are relatively inexpensive and lead to low background currents and broad potential windows [22,23]. Apart from the working electrode, an oriented immobilization of the antibody on the surface of an immunosensor is another crucial factor that needs to be considered in order to increase the sensitivity and specificity of immunosensors [24]. A number of procedures for oriented immobilization of biomolecules are available; among these strategies, the streptavidin/biotin system is an effective technique and has been widely used for enhanced sensitivity. Thus, this study aimed to develop an electrochemical immunosensor using streptavidin-modified SPCEs for the detection of the dengue NS1 antigen.

## 2. Material and Methods

### 2.1. Reagents and Chemicals

Bovine serum albumin (BSA), potassium chloride (KCl), sodium carbonate (Na_2_CO_3_), streptavidin, and sodium hydrogen carbonate (NaHCO_3_) were purchased from Sigma (St. Louis, MO, USA). Lightning-link horseradish peroxidase (HRP), Lightning-link biotin and an antibody concentration and clean-up kit were purchased from Innova Biosciences (Cambridge, UK). Dengue virus NS1 glycoprotein was acquired from Abcam (Cambridge, UK). Dipotassium hydrogen phosphate (K_2_HPO_4_), potassium dihydrogen phosphate (KH_2_PO_4_), sodium chloride (NaCl), and ferricyanide [Fe(CN)_6_]^3−^ were obtained from MERCK (Darmstadt, Germany). Carboxymethyldextran (CMD) (500,000 MW) was purchased from Fluka (Gillingham, UK). Two different clones (capture and detection) of monoclonal anti-NS1 antibody were purchased from ICL lab (Portland, OR, USA). Ready-to-use TMB (3,30,5,50-tetramethylbenzidine) substrates was purchased from Promega (Madison, WI, USA). BupH MES Buffered Saline Packs was purchased from Thermo scientific (Rockford, IL, USA), and Panbio Dengue Early kit was purchased from Panbio Diagnostics (Brisbane, Australia). Glycine was purchased from Biorad (Hercules, CA, USA).

The phosphate buffer saline (PBS) (0.01 M; pH 7.4) and carbonate buffer (0.1 M; pH 9.5) were prepared using ultra-pure water (UPW) obtained from a PURELAB Option Q-7BP MK 1 purification system (ELGA, Lane End, UK). BSA was prepared by dissolving 3% bovine serum albumin in PBS buffer (0.01 M; pH 7.4). Prior to the conjugation of the anti-NS1 antibody with HRP, sodium azide was removed from the antibody by using an antibody concentration and clean-up kit. The azide removal was performed according to manufacturer’s instructions. Subsequently, the NS1 detection antibody was conjugated using a Lightning-link horseradish peroxidase kit. Similarly, the conjugation procedure was performed according to manufacturer’s instructions.

### 2.2. Apparatus

SPCEs used in this study were purchased from a local company (ScrintTechnology, Penang, Malaysia). The SPCEs consisted of a three-electrode configuration (15 mm × 30 mm), which comprised a round-ended working electrode (4 mm in diameter), counter electrode, and silver pseudoreference electrode printed on a polycarbonate support. A ring-shaped insulating layer around the round-ended working electrode (8 mm × 8 mm) with a capacity of 100 µL was incorporated onto the SPCEs as an electrochemical cell (reservoir). Voltammetric and chronoamperometric studies were performed with an Autolab PGSTAT III potentiostat/galvanostat (Eco Chemie, Utrecht, The Netherlands) and interfaced to Nova 1.6 software. The scanning electron microscopy (SEM) images were obtained from Quanta FEG 450 (FEI, Eindhoven, The Netherland) at an acceleration voltage of 10 kV and a working distance of 10 µm.

### 2.3. Clinical Samples

In the current study, samples from suspected dengue patients presenting to the outpatient clinic in Hospital Universiti Sains Malaysia and sent to the Serology Laboratory, Department Medical Microbiology and Parasitology, Universiti Sains Malaysia (USM) were collected. Subsequently, those samples were tested for the dengue NS1 antigen using a Panbio dengue early ELISA kit. Afterwards, 10 positive and 10 negative dengue NS1 antigen samples were selected for this study. This study has received approval from the USM Human Ethics Committee (USM/JPeM/270.4. (1.3)).

### 2.4. Immobilization of the Anti-NS1

In this study, two techniques for the monoclonal anti-NS1 capture antibody immobilization on the carbon working electrode were investigated and compared: passive adsorption and covalent immobilization. Passive adsorption was conducted by coating the working electrode with 20 µL (10 µg/mL) of anti-NS1 monoclonal antibody prepared in carbonate buffer pH 9.5. Subsequently, the SPCEs were incubated overnight at 4 °C under controlled humidity. Later, non-specific bindings were blocked by incubating the electrode surface with 50 mmol/L glycine solution for 15 min.

For antibody-oriented immobilization, the carbon working electrode was pre-treated with carboxymethyldextran in order to introduce a carboxylic (COOH) group to the SPCE’s surface. For this purpose, 20 µL of 50 mg/mL CMD prepared in deionized water was added to the carbon working electrode and left overnight at room temperature. After washing with PBS, an equal volume of EDC-NHS (0.4 M EDC and 0.1 M NHS prepared in MES buffer pH 4.7) was then placed on the working electrode surface for 10 min at room temperature. Subsequently, 20 µL of 50 µg/mL of streptavidin was incubated for 1 h. After washing, 20 µL/mL of biotinylated anti-NS1 (10 µg/mL) prepared in 0.01 M PBS was incubated on the electrode surface for 1 h. Excessive ester groups on the streptavidin-treated SPCES were then blocked with 100 µL of 1 M ethanolamine chloride and incubated for 10 min in the dark.

### 2.5. Immunosensor Response to NS1

After successful immobilization of the monoclonal anti-NS1 capture antibody via passive adsorption and streptavidin methods on the SPCEs, various dilutions of full-length recombinant NS1 antigen prepared in 0.01 M PBS were added onto the electrode’s surface and left to react with the anti-NS1 capture antibody for 1 h. The assay was then completed by pipetting 20 µL of (5 µg/mL) HRP-labeled monoclonal anti-NS1 detection antibody. Finally, the electrodes were washed prior to electrochemical measurement.

Following various optimization procedures, the immunosensor responses were then evaluated using real serum samples. The serum samples were diluted in a buffer (1:2 ratio) and incubated on the anti-NS1 streptavidin/biotin-SPCEs. The assay was then completed as described above using HRP-labeled anti-NS1 detection antibody. In addition, a two-fold serial dilution of the serum sample was also evaluated in this study.

### 2.6. Measurement Procedure for Electrochemical Responses

All the electrochemical responses were performed at room temperature (27 ± 1 °C). Electrochemical measurements were carried out by placing a 70 µL TMB solution onto the electrode, covering the three electrodes’ area. A fixed reduction potential of −200 mV was applied to the reference electrode to measure the electrochemical responses resulting from the catalysis of H_2_O_2_ by the HRP enzyme. Each measurement was carried out in triplicate. Cyclic voltammetric measurements were carried out by scanning at 100 mV·s^−1^ and potential ranging from −600 mV to 1000 mV.

## 3. Results

### 3.1. Reproducibility and Stability of SPCEs

The reproducibility of carbon electrodes was characterized by performing cyclic voltammetry (CV) using a potassium ferricyanide redox system. The cyclic voltammogram data obtained from triplicate assays showed a relative standard deviation (RSD) value of 3.22%, suggesting a good reproducibility of the proposed immunosensor.

**Figure 1:**
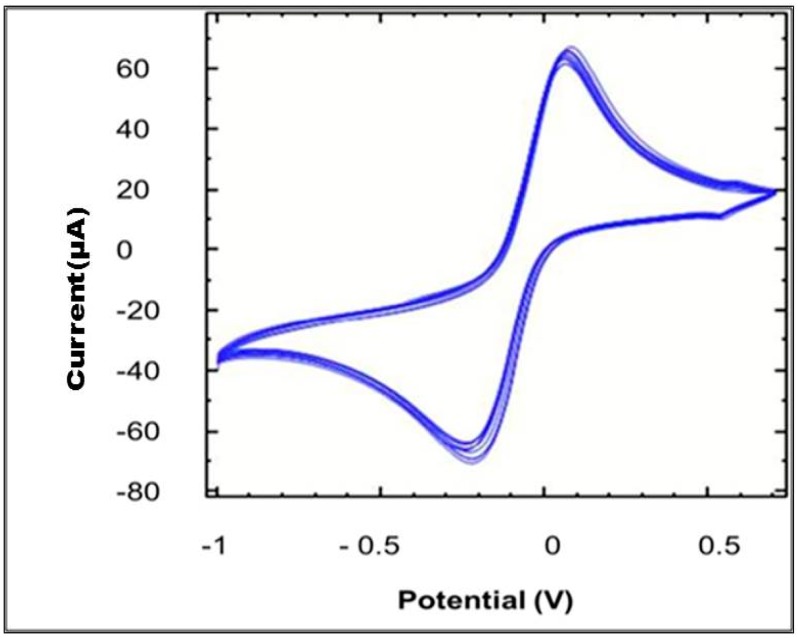
Cyclic voltammogram profile of the SPCEs with 10 cycles. Scanning was performed in 5 mM ferricyanide prepared in 0.1 M KCl at the scan rate of 100 mV/s.

Another critical parameter that was investigated was the stability of the SPCEs. In this study, the SPCEs were subjected to 10 cycles of CV at 100 mV/s scan rate in 5 mM ferricyanide solution prepared in 0.1 M KCl. As shown in Figure 1, a pair of well-defined redox peaks was observed after each cycle. A RSD value of 4% showed that the electrode was stable enough for electrochemical analysis.

### 3.2. SEM Characterization

**Figure 2:**
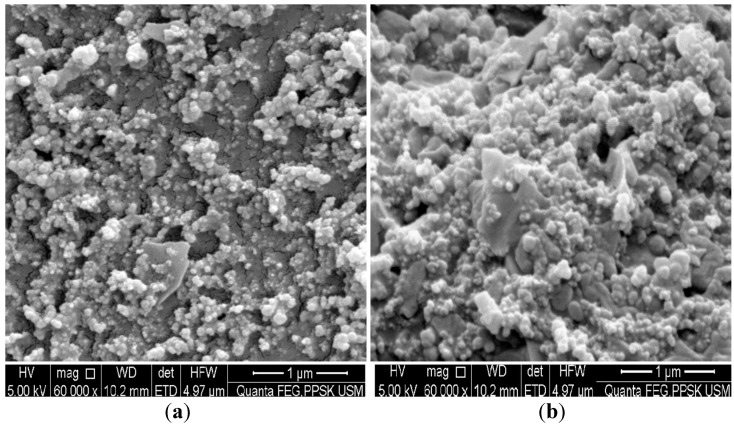
SEM surface images of (**a**) bare carbon electrode and (**b**) carbon electrode modified with antibody.

SEM was the first technique employed to visualize the electrode surface modification following antibody immobilization on the carbon surface. Figure 2a shows the SEM image of a bare working electrode surface. However, the surface was then covered with cloudy clusters (Figure 2b) following incubation with the anti-NS1 capture antibody.

### 3.3. Immobilization of the Anti-NS1

**Figure 3:**
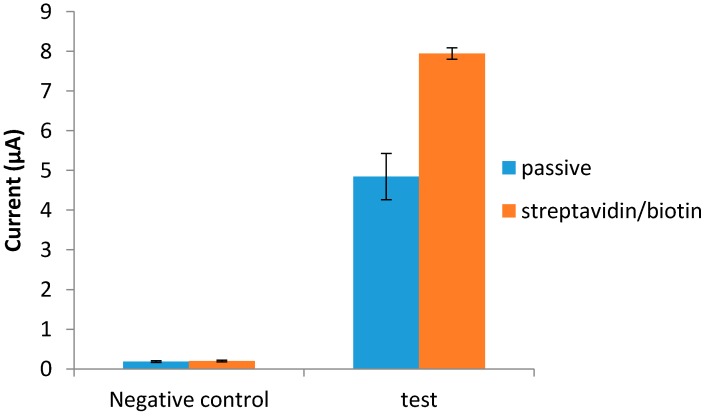
Immobilization of anti-NS1 capture antibody via passive adsorption and streptavidin/biotin system.

Two antibody immobilization techniques were tested, *i.e.*, passive adsorption and the streptavidin/biotin system, to improve the sensitivity of the developed immunosensor. Both techniques were found to be stable for the whole duration of the assay. Comparatively, the streptavidin/biotin technique yielded better signals than the passive adsorption technique (Figure 3).

**Figure 4:**
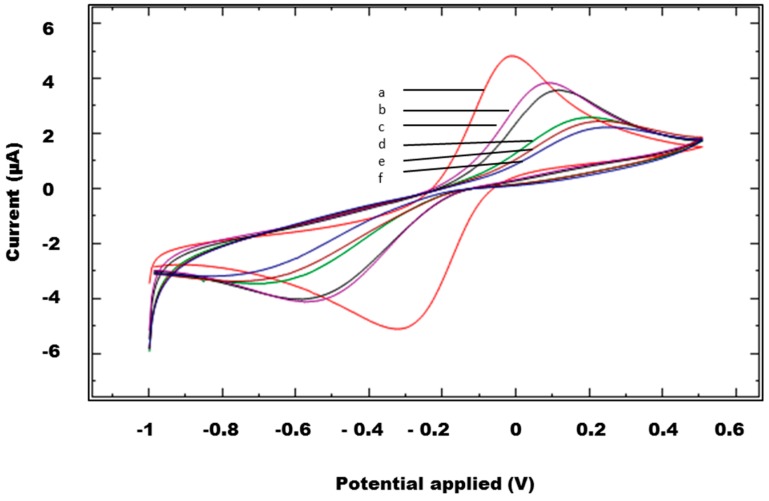
Cyclic voltammetries of the unmodified and modified SPCEs: (**a**) unmodified SPCEs, (**b**) SPCEs/CMD, (**c**) SPCEs/CMD/streptavidin, (**d**) SPCEs/CMD/streptavidin/biotinylated anti-NS1capture antibody, (**e**) SPCEs/CMD/streptavidin/biotinylated anti-NS1 capture antibody/glycine, (**f**) SPCEs/CMD/streptavidin/biotinylated anti-NS1capture antibody/glycine/NS1 antigen.

An indirect method was also employed to examine the immobilization of the anti-NS1 capture antibody and other immunoreagents used in the immunosensor. The stepwise immobilization of the anti-NS1 capture antibody and other immunoreagents on SPCEs was accomplished by CV using 5 mM [Fe(CN)_6_]^3−^ prepared in 0.1 M KCl solution as the redox probe. A decrease in the anodic and cathodic peaks was observed when the SPCEs were modified with the anti-NS1 capture antibody (Figure 4). A similar redox behavior was observed when other immunoreagents were immobilized on SPCEs.

### 3.4. Optimization of Immunoreagents

**Figure 5:**
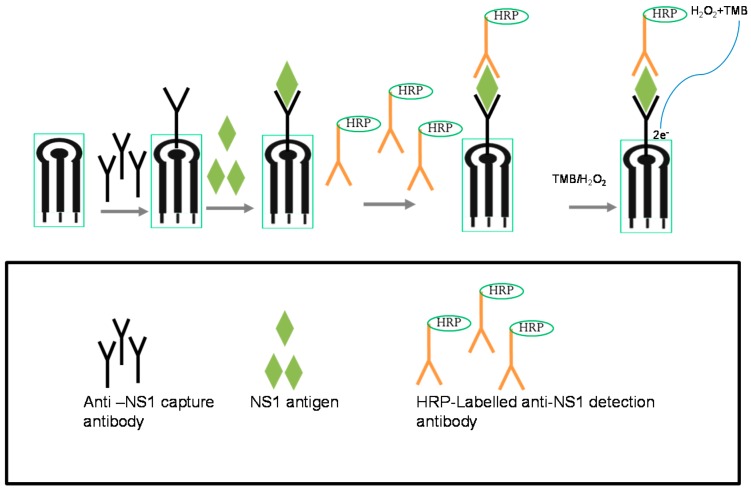
Development of dengue NS1 immunosensor.

The electrochemical immunosensor system developed for NS1 detection in this study was based on a direct sandwich ELISA format with HRP used as the enzyme label and TMB/H_2_O_2_ as the substrate/mediator system (Figure 5).

**Figure 6:**
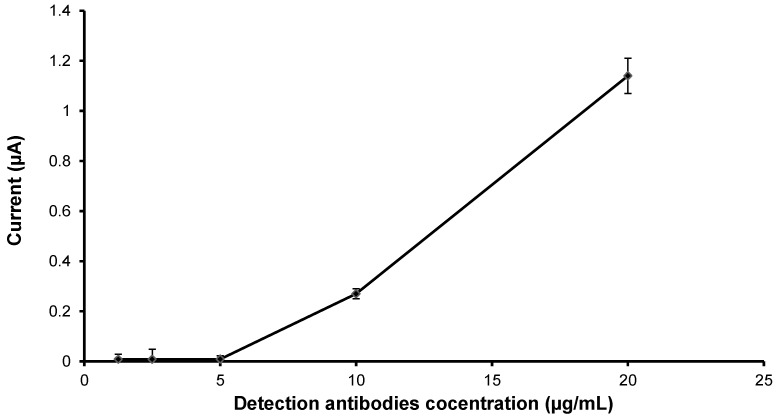
Optimization of detection anti-NS1 antibody concentration.

In order to reduce background signals, various optimizations were performed. First, the optimal concentration of anti-NS1 detection antibody needed was investigated. In this study, SPCEs were first blocked with 50 mmol/L glycine for 15 min. Subsequently, the SPCEs were incubated with various concentrations of HRP-conjugated anti-NS1 detection antibody (0 to 20 µg/mL), followed by electrochemical measurement. The background current signal of the detection antibody is shown in Figure 6. Concentration above 5 µg/mL produced background current signals; the highest amount of detection antibody gave the highest background signal. Thus, for subsequent assays, an antibody concentration of 5 µg/mL was chosen.

**Figure 7:**
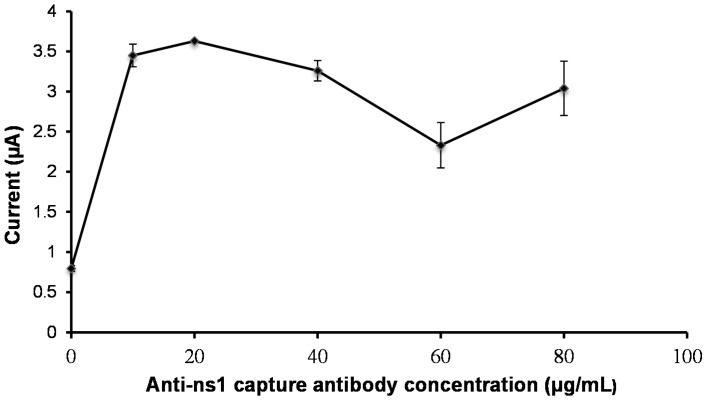
Optimization of the capture antibody concentration.

Following this, the concentration of anti-NS1 capture antibody was optimized. To achieve this, different concentrations of capture anti-NS1 antibody ranging from 10 to 80 µg/mL were immobilized on the working electrode. As shown in Figure 7, the current peak increased as the antibody concentration increased. A maximum current response was acquired when an antibody concentration of 20 µg/mL was used.

**Figure 8:**
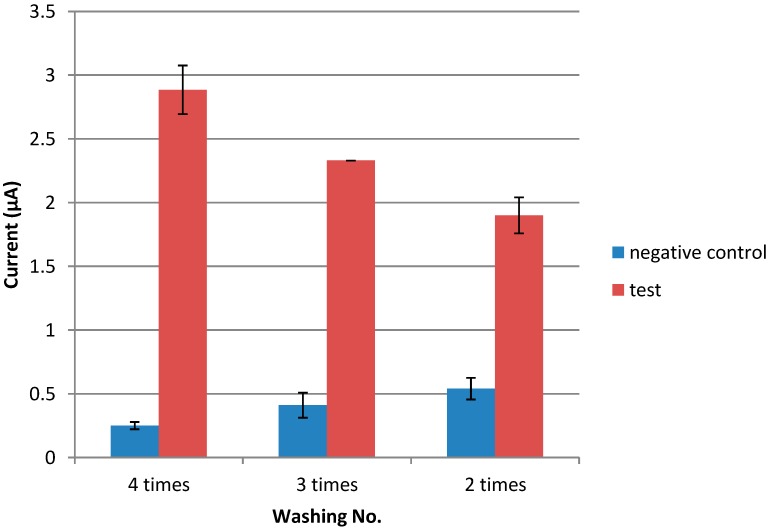
Optimization of washing frequency.

The incubation period for CMD, streptavidin, anti-NS1 capture antibody, blocking agent, NS1 antigen, and anti-NS1 detection antibody were subsequently determined. Incubation periods of 15, 30, and 60 min were tested for each step of the assay. It was found that the optimal incubation periods for streptavidin, the anti-NS1 capture antibody, the blocking agent, the NS1 antigen, and the detection antibody were 15, 60, 60, and 60 min, respectively (data not shown). For CMD, overnight incubation was found to be the most suitable period (data not shown). Finally, the optimum washing frequency was also studied. As shown in Figure 8, abundant washing (four times) produced good signals with a low background response.

### 3.5. Electrochemical Response of the Immunosensor

**Figure 9:**
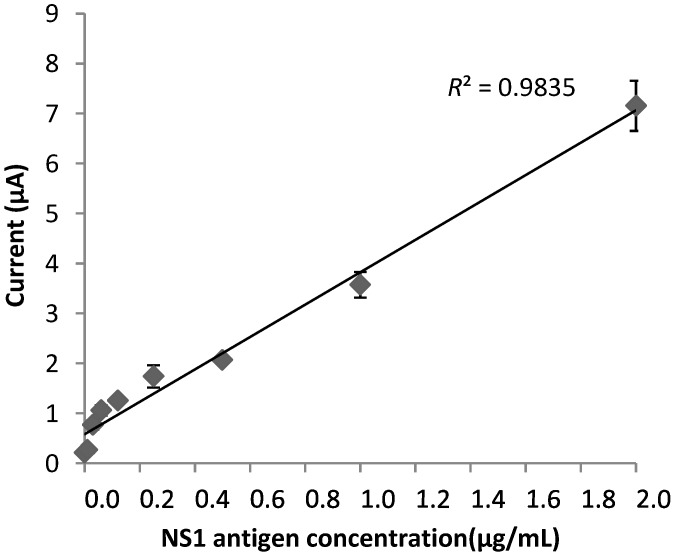
Calibration curve of the immunosensor for the detection of NS1 protein.

A direct sandwich ELISA format based experiment using the optimized parameters was then performed to analyze the sensitivity of the assay. SPCEs were incubated with various concentrations of recombinant NS1 antigen, ranging from 2 to 0.01 µg/mL. The electrochemical response generated by the catalytic reaction between H_2_O_2_ and HRP-conjugated anti-NS1 was measured using chronoamperometry. The immunosensor showed a linear response between 0.5 and 2 µg/mL (Figure 9), with a detection limit of 0.03 µg/mL.

### 3.6. Detection of NS1 Antigen in Real Serum Samples

**Figure 10:**
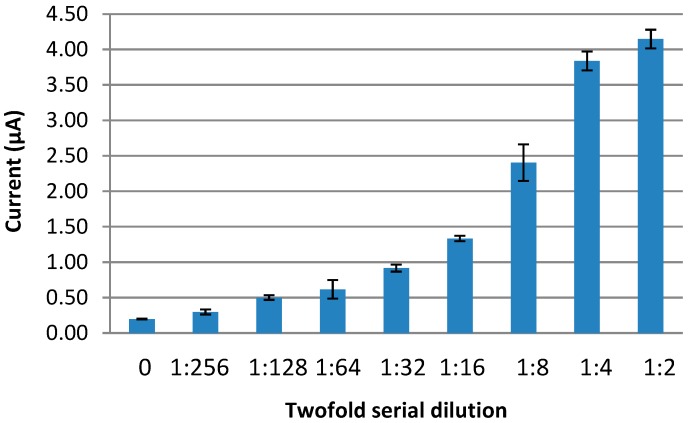
Optimization of serum dilution for the detection of dengue NS1.

The proposed immunosensor was also tested against real NS1 samples. However, firstly, the optimum serum dilution needed to be determined. To achieve this, a twofold serial dilution of pooled NS1 positive serum was prepared and subjected to the NS1 antigen immunosensor. It was found that the immunosensor responses for the NS1 positive serum pool were detectable up to 1:128 dilution and the developed sensor yielded a maximum response at 1:2 dilution (Figure 10).

**Figure 11:**
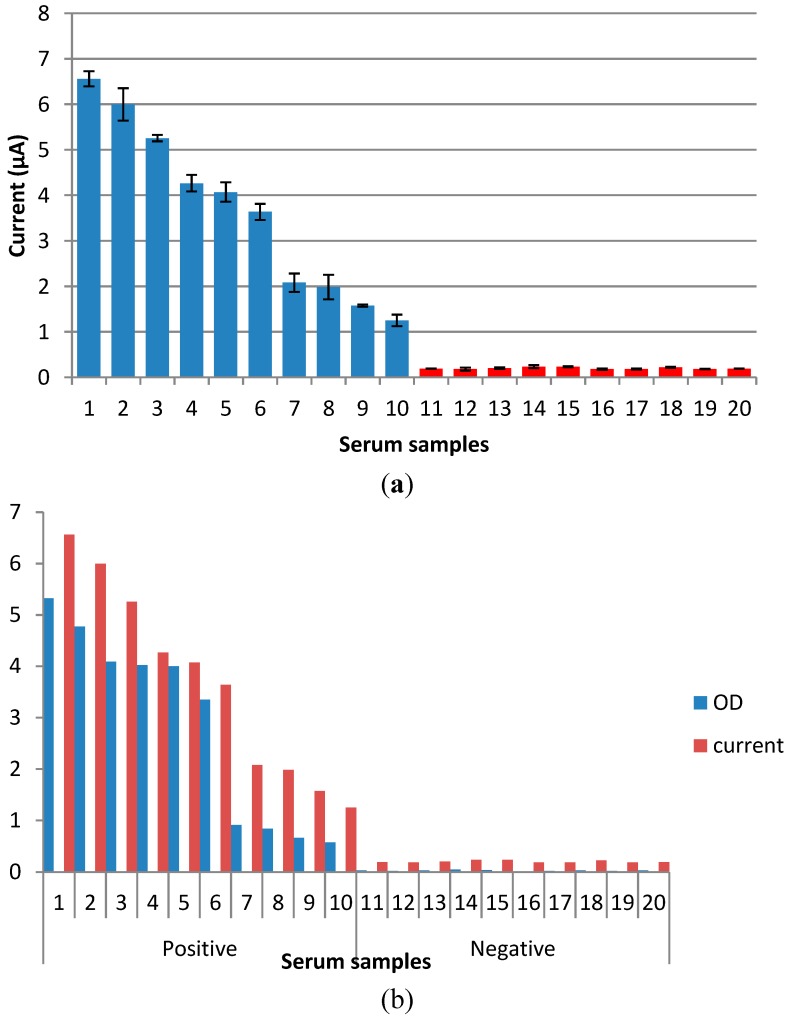
(**a**) Dengue NS1 detection in real samples; (**b**) Comparative analysis of dengue NS1 serum samples performed with Panbio ELISA kit and immunosensor.

Afterwards, the 10 positive and 10 negative dengue NS1 serum samples were diluted 1:2 and tested. Results based on these 20 samples showed that this immunosensor could successfully detect all 10 positive and 10 negative dengue NS1 serum samples (Figure 11a). Following this, the sensitivities of each individual sample obtained via immunosensor and Panbio ELISA kit (in the form of optical density (OD)) (Figure 11b) were compared and it was found that samples with a high OD produced a higher current signal in the immunosensor assay; the opposite was true for samples with lower OD.

## 4. Discussion

The application of SPCEs is not merely useful in terms of cost-effectiveness; it also yields reproducible results and offers a more sensitive method for the detection of the target analyte. Thus, reproducibility of SPCEs was also evaluated in this study. The relative standard deviation of the measurements for the triplicate electrodes was found to be 3.28%, suggesting a good reproducibility for the proposed immunosensor, attributable to the carbon ink used in the fabrication of SPCEs. In order to measure the electrochemical response, the SPCEs are subjected to certain potential for a given time. Therefore, stability is a crucial factor in producing good results without affecting the conductivity of the surface for a given time [19]. In this study, SPCEs were subjected to 10 cycles of CV by using the ferricyanide redox system. The cyclic voltammograms of 10 superimposed cycles showed good similarity with each other. Therefore, the continuous flow of electrons over 10 cycles demonstrated that the electrode was stable for the assay. This stability could be attributed to the strong binding of carbon paste to a ceramic surface.

Oriented immobilization of antibodies plays an indispensable role in the fabrication of electrochemical immunosensors [25]. In this study, comparative immobilization methods for capture antibody immobilization were studied, *i.e.*, passive adsorption and the streptavidin/biotin system. Both methods yielded stable responses during the assay, although the streptavidin/biotin system showed better detection signals than the passive technique. This improvement in signal detection with the streptavidin/biotin system was perhaps due to the availability of more antibody recognition sites caused by the oriented antibody immobilization. The passive adsorption was less effective due to the inconsistent orientation of the anti-NS1 capture antibody. When antibody molecules are passively adsorbed onto surfaces, the antibody stabilizes and binds weakly to the surface through electrostatic, hydrophobic, and polar intermolecular interactions, resulting in a random orientation of the paratopes and significant loss of antibody activity [26,27].

The antibody immobilization could be visualized through SEM images. The SEM images in the current study showed that the non-modified SPCEs displayed empty spaces on the surface. On the contrary, SPCEs modified with the anti-NS1 antibody exhibited cloudy clusters, suggesting that the antibody was successfully immobilized.

The immobilization of the capture antibody and other immunoreagents were further verified by the ferricyanide redox system with CV. Each time the SPCEs were modified with any of the immunoreagents, such as CMD or anti-NS1 antibody, a decrease in the anodic and cathodic peaks was observed. This decrease in the area of redox peak was due to the adsorption of the NS1 antibody and other immunoreagents that are insulating in nature, therefore producing resistance to the flow of electrons or causing interference in the flow of electrons, eventually resulting in the decrease of redox peaks [28,29].

The analytical performance of an immunosensor is dependent on various parameters of the assay procedures, including washing frequency, incubation time, and optimum concentration of capture antibody and detection antibody [30]. In order to enhance the performance of the developed NS1 antigen immunosensor, all these parameters were investigated. An initial optimization procedure attempted to reduce background signals, which may lead to false positive results. The optimization assay for the anti-NS1 detection antibody showed that a concentration above 5 µg/mL produced false background signals, probably because an excessive amount of antibody may breach the blocking barrier and could lead to false results. Hence, a concentration of 5 µg/mL of anti-NS1 detection antibody was chosen for subsequent experiments. Optimizing the concentration of anti-NS1 capture antibody is also a crucial part of developing this immunosensor, as it directly affects the sensitivity of the assay [16]. It was demonstrated in this study that the use of 20 µg/mL anti-NS1 capture antibody gave the best current response, suggesting that at this concentration there is maximum availability of antibody recognition sites on the electrode [31]. On the contrary, concentration below or above 20 µg/mL resulted in poor detection signals, most probably due to a lack of antibody recognition sites and saturated antibody, respectively [32].

In this study, incubation time for the immobilization and immunoreaction of streptavidin, the biotinylated anti-NS1 antibody, glycine, the NS1 antigen, and detection was found to be 60, 60, 15, 60, and 60 min, respectively. Additionally, it was determined in this study that four washes produced a high detection signal and fewer background signals. Extensive washing could remove more nonspecific agents, which could otherwise interfere in the immunoreaction between the antibody and antigen.

Following the completion of various optimization procedures, the immunosensor was subjected to sensitivity testing. For this purpose, a certain range of target NS1 antigen concentrations was chosen and tested to reflect the amount of NS1 antigen present in the real clinical samples. The developed NS1 antigen immunosensor had a detection limit of 0.03 µg/mL, which is less than previously reported by Wu *et al.* (2005) [33]. In real serum samples, the NS1 antigen could be detected at levels up to 15 μg/mL [34,35]. However, Alcon *et al.* (2003) [36] demonstrated that the dengue NS1 antigen may vary, depending on the type of infection. NS1 levels ranged from 0.04 to 2 μg/mL during primary dengue infection. This suggests that the developed biosensor in our study is capable of detecting dengue the NS1 antigen in real clinical serum samples during acute dengue infection. This was further supported and validated following the use of 20 real serum samples. The results obtained were parallel to the Panbio ELISA results and the proposed immunosensor could successfully differentiate between the NS1 positive and negative samples. This suggests that the developed immunosensor is able to detect dengue NS1 in real serum samples and has very good potential for the useful diagnosis of acute dengue infection.

## 5. Conclusions

In this study, we have proposed a simple approach to the development of an electrochemical immunosensor for the detection of the dengue NS1 antigen in real samples. This approach is based on the immobilization of capture antibodies via the streptavidin/biotin system on SPCEs. Consequently, the application of the streptavidin/biotin system in the electrochemical immunosensor resulted in well-oriented immobilization of the capture antibody, which in turn enhanced the sensitivity of the assay. SPCEs used in this study showed good reproducibility and stability, although the electrical conductivity decreased with the immobilization of immunoreagents. This electrical conductivity could be enhanced by using a more advanced electrode, which in turn will improve the detection signals.
